# Modified statistical dynamical diffraction theory: analysis of model SiGe heterostructures

**DOI:** 10.1107/S0021889813011308

**Published:** 2013-06-07

**Authors:** P. K. Shreeman, K. A. Dunn, S. W. Novak, R. J. Matyi

**Affiliations:** a College of Nanoscale Science and Engineering, University at Albany–SUNY, 257 Fuller Road, Albany, NY 12203, USA

**Keywords:** statistical dynamical diffraction theory, defective semiconductor heterostructures

## Abstract

A modified version of the statistical dynamical diffraction theory (mSDDT) permits full-pattern fitting of high-resolution X-ray diffraction scans from thin-film systems across the entire range from fully dynamic to fully kinematic scattering. The mSDDT analysis has been applied to a set of model SiGe/Si thin-film samples in order to define the capabilities of this approach. For defect-free materials that diffract at the dynamic limit, mSDDT analyses return structural information that is consistent with commercial dynamical diffraction simulation software. As defect levels increase and the diffraction characteristics shift towards the kinematic limit, the mSDDT provides new insights into the structural characteristics of these materials.

## Abstract 2

A modified version of the statistical dynamical diffraction theory has been applied to a set of model SiGe/Si thin-film samples in order to define the capabilities of this approach for returning structural information from defective semiconductor heterostructures.

## 1. Introduction

The ability to effectively characterize strain-relaxed structures such as silicon–germanium (SiGe) material commonly seen in modern thin-film technology is one of the on-going challenges in the semiconductor industry. High-resolution X-ray diffraction (HRXRD) is commonly used for such analyses, since this method offers a highly sensitive nondestructive approach that can be easily used to collect data from these materials. The analysis of HRXRD data is often based on the Takagi–Taupin equations (Takagi, 1962 ▶, 1969 ▶; Taupin, 1964 ▶) or their derivatives. The Takagi–Taupin (T–T) equations are in turn based on the dynamical theory of X-ray diffraction, which assumes that the structures are crystallographically perfect (or nearly so), with very small fluctuations of lattice displacements.

The use of this approach for the analysis of highly defective layers is not recommended, however. For example, fully relaxed silicon–germanium epitaxial layers grown with high Ge concentrations on silicon substrates are very poorly described using the dynamical diffraction theory (Shreeman & Matyi, 2010 ▶), as is structurally defective ion-implanted SiGe with a range of germanium concentrations (Shreeman & Matyi, 2011 ▶). A purely dynamical analysis strategy is not appropriate for the analysis of these cases of fully or partially relaxed samples.

An approach that has consistently shown promise for the analysis of structurally defective materials is the statistical dynamical diffraction theory (SDDT). It was first devised by Kato (1980*a* ▶,*b* ▶) and further developed by others, including Bushuev (1989*a* ▶,*b* ▶), Punegov (1990*a* ▶,*b* ▶, 1991 ▶, 1993 ▶), Punegov & Kharchenko (1998 ▶) and Pavlov & Punegov (2000 ▶). The typical approach in the SDDT is to integrate incoherent scattering (due to defects) and coherent scattering (dynamical-based from crystallographically perfect structures) using two parameters: a static Debye–Waller factor (*E*) and a complex correlation length (τ). On the basis of the Bushuev treatment, we have a set of dynamical T–T equations given by
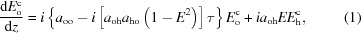


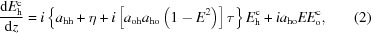
where 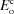 and 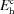 are the coherent (dynamic) amplitudes, η is the simplified deviation parameter, and the coefficients *a_ij_* are Bushuev’s (1989*a* ▶,*b* ▶) definitions of susceptibility given by
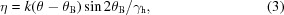


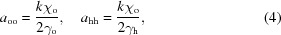


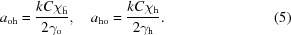
Here *k* is the wavevector, γ_h_ is the direction cosine of the diffracted beam, *C* gives the polarization of the incident X-rays, and χ_o_ and χ_h_ are the usual electrical susceptibilities. The specific procedures of statistical averaging are explained in detail by Bushuev (1989*a* ▶,*b* ▶) and are also discussed elsewhere (Li *et al.*, 1995 ▶; An *et al.*, 1995 ▶; Punegov, 1990*a* ▶,*b* ▶, 1991 ▶, 1993 ▶; Pavlov *et al.*, 1995 ▶; Punegov & Kharchenko, 1998 ▶; Pavlov & Punegov, 2000 ▶; Authier, 2001 ▶).

The two new parameters τ and *E* were introduced by Kato (1980*a* ▶). The long-range-order parameter (*E*) is called the static Debye–Waller factor; *E* ranges from a value of unity for a fully strained (dynamical) structure to zero for the fully relaxed (kinematically limited) case. The second factor is the short-range complex correlation length τ, which characterizes the scale over which definite spatial relationships between lattice sites can be considered applicable in a defective crystal (Authier, 2001 ▶). The magnitude of this quantity can be complex, since it is affected by phase relationships in the lattice as well as distance and thus presents a number of mathematical difficulties. Fortunately, a mosaic block model based on the method of Bushuev (1989*b* ▶) has been found to provide a useful simplified approach for incorporating this parameter. As discussed by Shreeman & Matyi (2010 ▶) we can use the Bushuev mosaic block model and consider only the real part of τ by using
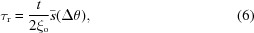
where 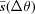 = 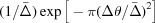 and 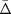 = 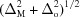 (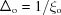 with 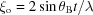). In these expressions, Δ_o_ is the width of the reflection of the individual blocks, Δ_M_ is the width of the angular distribution of the mosaic blocks (assumed to be Gaussian), and *s* represents the convolution of the individual mosaic block diffraction and the angular distribution of the blocks.

The modified T–T equations are part of the coherent scattering calculation within the SDDT framework. Typically, the SDDT model assumes that the substrate diffraction profile remains perfectly dynamical. We have found, however, that broadening effects due to the presence of defects in the substrate will have an impact on the diffracted amplitude and need to be incorporated into the SDDT model. This essential modification of the SDDT is detailed in recent work (Shreeman & Matyi, 2010 ▶, 2011 ▶; Shreeman, 2012 ▶). In this approach the basic definition 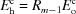 is used, where the reflection coefficient from a given layer is defined in terms of the amplitude emerging from the material beneath. We can revise this term by instead incorporating a broadening effect (*B*_e_) by defining 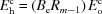, in which
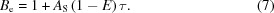
Here *A* is a normalization factor with respect to the substrate peak. As discussed in detail by Shreeman (2012 ▶), this modification explicitly addresses the impact of structurally defective layers on the scattered coherent amplitude and the resultant observed intensity distribution from an otherwise dynamically diffracting substrate in a layer-on-substrate materials system.

The preceding discussion has considered only the coherent (dynamic) component of the diffracted intensity. As discussed elsewhere (Shreeman & Matyi, 2010 ▶), the incoherent or kinematic component is described by the energy transfer equations
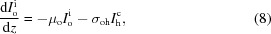


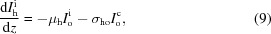
where the superscripts i and c refer to the incoherent and coherent contributions in the incident (o) and diffracted (h) beam directions, μ is the photoelectric absorption coefficient (divided by the direction cosine γ_*i*_), and 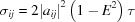 is the diffuse scattering kinematic cross section (Kato, 1976*a* ▶,*b* ▶; Bushuev, 1989*a* ▶,*b* ▶). The total intensity is given by the sum 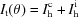, where 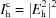.

We refer to the incorporation of equation (7) into (1) and (2) as the modified statistical dynamical diffraction theory (mSDDT). It offers a method of incorporating broadening of the substrate peak using the same parameters (*E* and τ) employed for layer peak broadening. In the present study, we have examined a set of well characterized model samples; the analysis of these materials should illustrate the utility of mSDDT for providing characterizations of partially and fully strain relaxed thin-layer materials.

## 2. Experimental

Samples of SiGe were grown at approximately 823 K by molecular beam epitaxy, with Table 1 ▶ illustrating the experimental design. The first set (denoted SiGe-2, -3 and -4) had a nominal consistent thickness (40 nm) with varying targeted Ge compositions ranging from 25 to 75%. In contrast, the second set (SiGe-a, -b and -c) had nominally constant composition (approximately 50% Ge) with thickness ranging from 20 to 70 nm. With these parameters, a full range of pseudomorphic strain behavior – from fully strained to fully relaxed – was sought.

High-resolution X-ray analyses were performed using a Bruker D8 diffractometer equipped with an Eulerian quarter circle, a graded parabolic mirror and a two-crystal four-reflection [symmetric Ge(220)] monochromator. Supporting characterizations were performed using secondary-ion mass spectrometry (SIMS) and transmission electron microscopy (TEM). TEM specimens were prepared in an FEI Nova Nanolab 600 focused ion beam/scanning electron microscope equipped with a Pt gas injection system for the deposition of surface protection layers and an OmniProbe nanomanipulator used for *in situ* liftout. Diffraction contrast and lattice images were recorded in a Jeol 2010F field emission transmission electron microscope operated at 200 kV. SIMS analyses were performed with an IonTof V-300 spectrometer with Cs^+^ bombardment. Analyses were performed with point-to-point normalizing to Si^2−^ ions, a procedure that has been shown to minimize so-called matrix effects. Standard SiGe layers that were close in composition to the current materials and which had been previous calibrated by Auger electron spectroscopy and Rutherford back scattering spectrometry were used to determine layer composition.

## 3. Results

For clarity, we will consider the experimental results based on the nominal structural characteristics of the sample sets, namely constant-thickness *versus* constant-composition samples.

### 3.1. Constant-thickness samples

**Figure 1:**
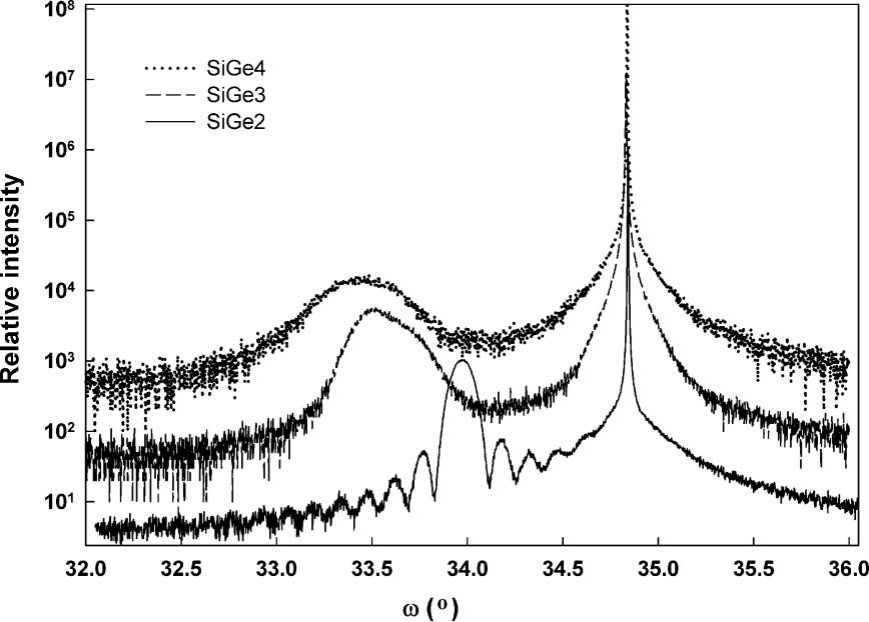
Experimental 004 diffraction scans from the constant-thickness sample set.

Fig. 1 ▶ illustrates symmetric 004 scans obtained from the samples with a nominally fixed thickness but a variable Ge concentration. Sample SiGe-2 appears to be fully strained and exhibits the typical signs of a dynamically diffracting thin-film epitaxial material system: a sharp substrate reflection, a relatively narrow layer peak and well defined interference fringes. In contrast, the diffraction scan from sample SiGe-3 showed a strong nondynamical layer diffraction peak while exhibiting no subsidiary dynamical fringes. The layer peak seen in the diffraction scan from sample SiGe-4 was consistent with a highly relaxed layer with an even broader and lower-intensity layer peak. SIMS analysis from samples SiGe-2 and SiGe-3 showed that the nominal layer thickness of 40 nm and the nominal concentrations of 25% Ge and 50% Ge were reasonable estimates for these samples.

All of the X-ray data from the constant-thickness sample set were then fitted by the mSDDT method. In our current implementation, the fitting process was performed by calculation of a trial curve followed by a visual comparison against an experimental diffraction profile and subsequent manual adjustment of the input structural model. For mathematical simplicity, the background was incorporated from a scan of a perfect single-crystal silicon standard sample. We recognize that these approaches are inferior to a more sophisticated automated process for optimization of a structural model *via* minimization of the error between the experimental and calculated profiles (using, for instance, a Levenberg–Marquardt fit optimization algorithm). However, for our current purposes of exploring the utility of the mSDDT approach and benchmarking its performance with technologically relevant materials, a manual fitting procedure was found to be acceptable.

**Figure 2:**
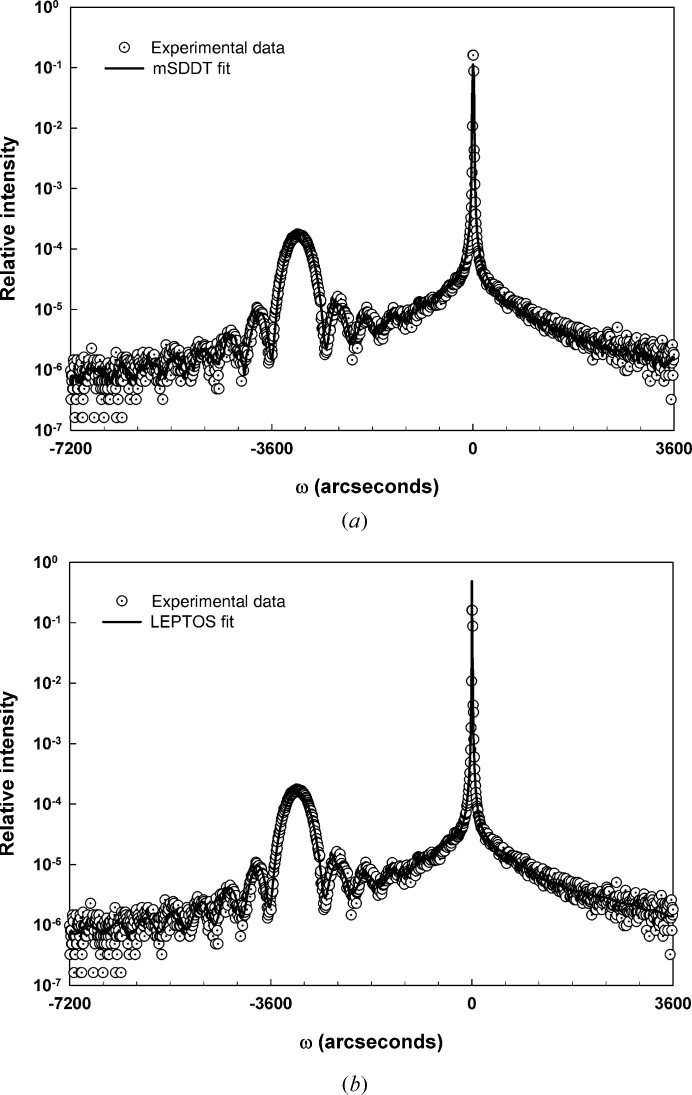
Fit results for SiGe-2: (*a*) mSDDT fit, (*b*) *LEPTOS* fit.

Fig. 2 ▶(*a*) illustrates the 004 scan from sample SiGe-2 along with the fit obtained with the mSDDT approach. In calculating the fit the composition was fixed at 25% Ge. Pseudomorphic strain (∊) was incorporated into the fit by calculating a relaxed lattice parameter at the assumed composition and then calculating the strain based on the final fitted lattice parameter. Additionally, the fit was achieved using a long-range-order parameter *E* equal to unity and a value of Δ_M_ equal to zero, corresponding to a perfectly dynamically diffracting sample.

While the fit generated for sample SiGe-2 by the mSDDT approach appears to be reasonable, it was desirable to verify this result through comparison with a commercial dynamical fitting software package. Hence Fig. 2 ▶(*b*) illustrates the fit that was attained using a well known commercially available dynamical diffraction fitting package (*LEPTOS*, version 7.03; Bruker AXS GmbH, Karlsruhe, Germany). Although the *LEPTOS* package uses a recursive matrix formalism rather than the T–T equations, its proven capabilities for fully describing dynamically diffracting thin-film structures make it a useful tool for assessing the basic characteristics of the mSDDT approach developed here.

A qualitative comparison shows that the two fits (mSDDT and *LEPTOS*) give very similar results. Quantitatively, the thickness and composition values generated by the *LEPTOS* fit (40.4 nm and 28.3% Ge, respectively) are similar to the values (40 nm and 25% Ge) used for the mSDDT. The difference in the two composition values comes from the fact that the *LEPTOS* value was generated by an automatic fitting procedure based on a genetic algorithm, while the mSDDT analysis employed the nominal layer composition as input to a direct calculation with no fit minimization. The Δ*c*/*c* strain from *LEPTOS* (∊ = 23 × 10^−3^) was similar to the value (∊ = 24 × 10^−3^) returned by the mSDDT analysis.

**Figure 3:**
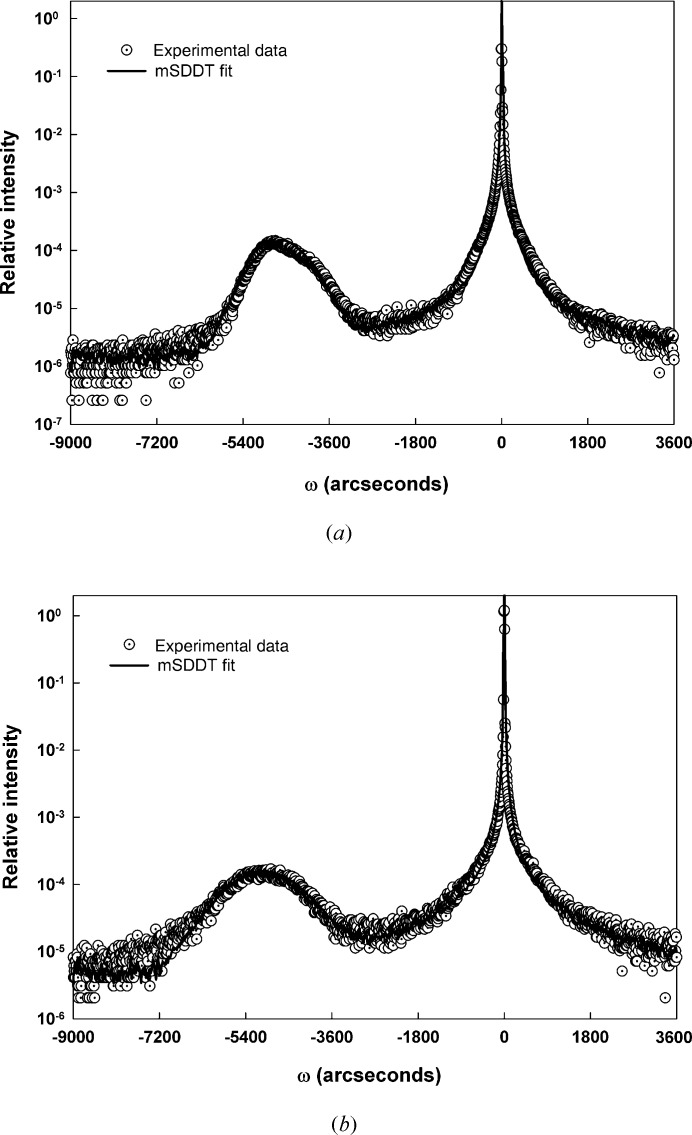
mSDDT fits from (*a*) SiGe-3 and (*b*) SiGe-4.

Fig. 3 ▶ shows the experimental 004 X-ray data and the subsequent mSDDT fits for samples SiGe-3 and SiGe-4. As mentioned above, these samples are no longer purely dynamically diffracting systems, so they are not accessible by conventional HRXRD analytical approaches. However, the mSDDT is not subject to this limitation. For SiGe-3, the composition was set at the nominal value of 50% Ge for the mSDDT fitting, and the nominal thickness of 40 nm was used. With these values in place the optimum fit returned with values of *E* = 0.25 and Δ_M_ = 5.5′′. As expected, these values indicate a diffraction characteristic that is intermediate between the purely dynamic and purely kinematic limits. Somewhat surprisingly, the strain that was returned by the mSDDT fit was ∊ = 25 × 10^−3^, which is almost identical to the value seen in the dynamically diffracting sample SiGe-2.

The experimental 004 X-ray scan and the resultant mSDDT fit from SiGe-4 where the composition and layer thickness were set at 75% Ge and 40 nm are also shown in Fig. 3 ▶. The fit of the experimental data is apparently quite reasonable, especially considering that the structure is defined as fully relaxed, and that it is fitted with only a single layer. The mSDDT analysis showed that this sample was essentially at the kinematical limit with the value of the static Debye–Waller factor *E* = 0.1. Further evidence of relaxation in this sample with high germanium content is found in the reduced value of strain (∊ = 11 × 10^−3^) and the increased value of Δ_M_ = 9′′.

### 3.2. Constant-composition set

The samples constituting the second set (SiGe-a through SiGe-c) are characterized by a nominally constant germanium composition of 50% Ge but thicknesses ranging from 20 to 70 nm. SIMS results from these three samples confirmed that the nominal compositions and thicknesses are reasonable assessments of the structural characteristics of these samples.

**Figure 4:**
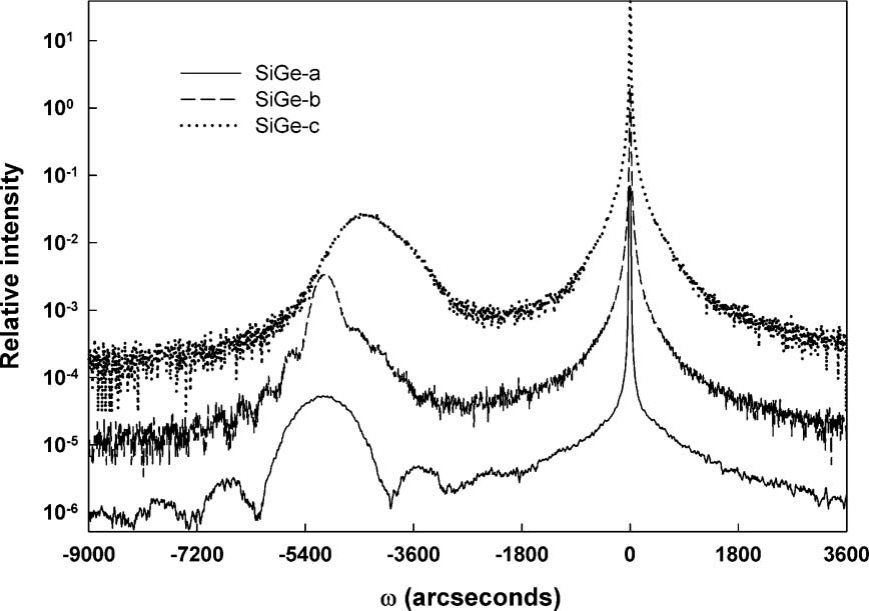
Experimental 004 diffraction scans from the constant-composition sample set.

**Figure 5:**
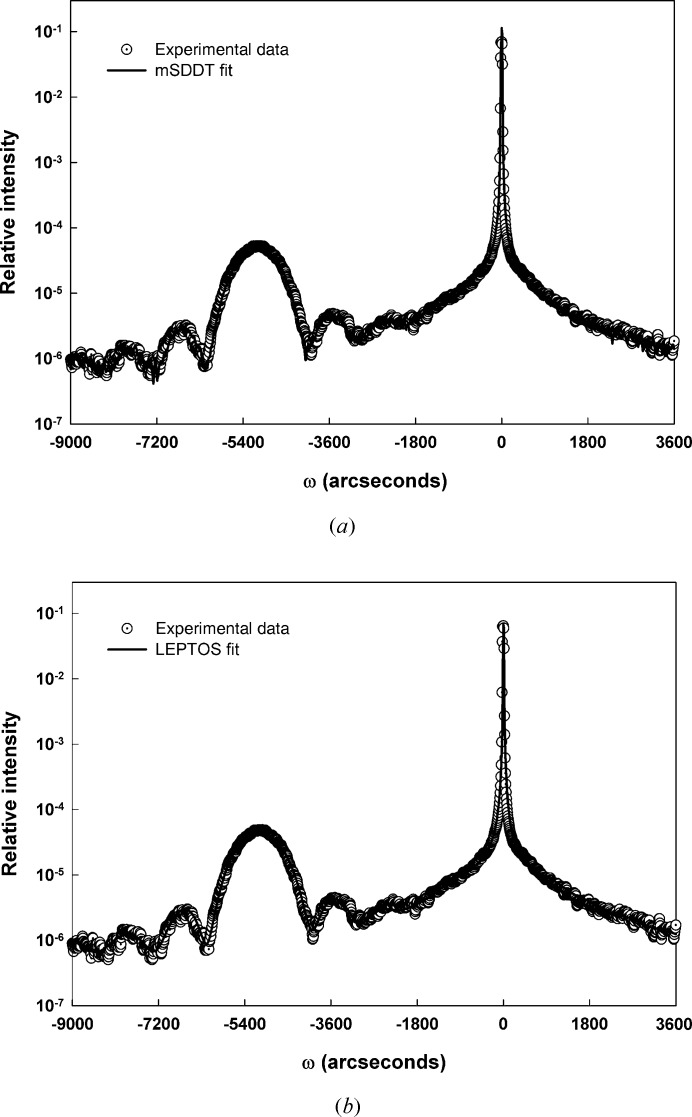
Fit results for SiGe-a: (*a*) mSDDT fit, (*b*) *LEPTOS* fit.

Fig. 4 ▶ shows the symmetric 004 diffraction scans that were generated by this second sample set. The experimental HRXRD scan from the thinnest (and presumably the most highly perfect) sample in this set shows that sample SiGe-a appears to diffract dynamically. The mSDDT fitting shown in Fig. 5 ▶(*a*) confirmed this by returning values of unity and zero for *E* and Δ_M_, respectively. Again, the apparent dynamically diffracting nature of this sample invites comparison and validation, so the corresponding fit to the data achieved with the commercially available *LEPTOS* software is shown in Fig. 5 ▶(*b*). Both fits are consistent with a layer thickness of 18.5 nm and a germanium content of 50% Ge and show similar values of strain (32 × 10^−3^ and 38 × 10^−3^ for mSDDT and *LEPTOS*, respectively).

**Figure 6:**
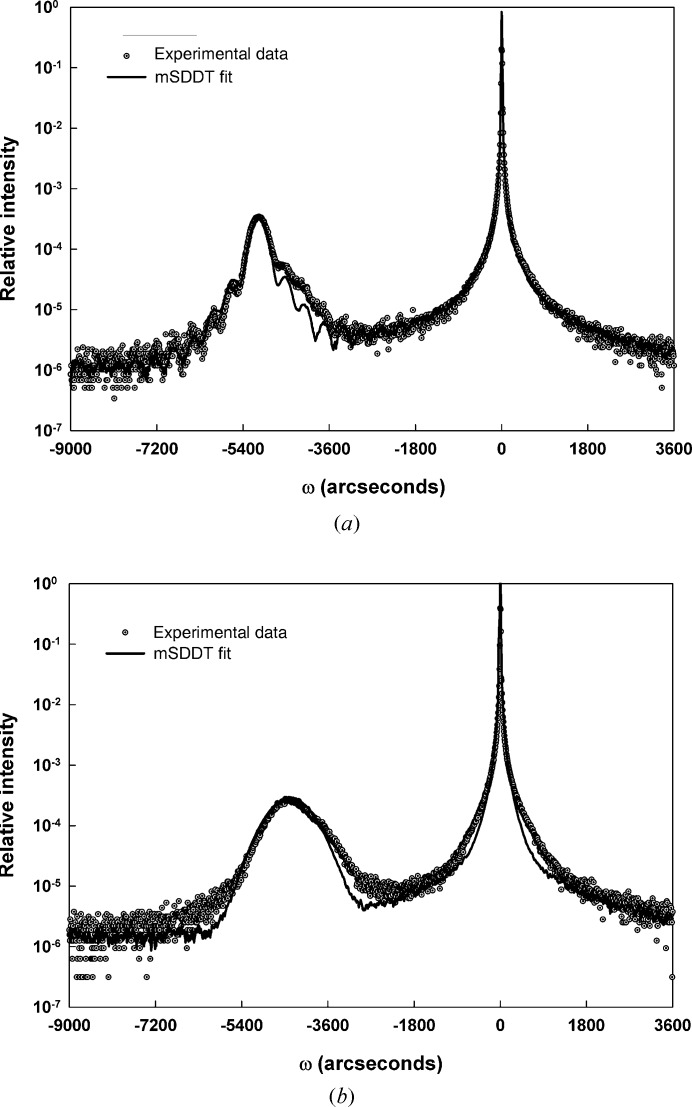
mSDDT fits from (*a*) SiGe-b and (*b*) SiGe-c.

Sample SiGe-c was observed to be similar to SiGe-3 in that it shows a single broad peak with no evidence whatsoever of dynamical fringes. Sample SiGe-b showed an intermediate behavior of exhibiting some dynamical fringes along with a broadened diffraction profile from the layer. Fig. 6 ▶ presents the mSDDT fits obtained for the thicker samples (nominally 50 and 70 nm) in the constant-composition set. Sample SiGe-b was notable because it shows what is apparently a superposition of dynamic and kinematic diffraction effects, with dynamic fringes visible on a broadened (presumably partially kinematic) layer peak. The mSDDT fit was performed using a single layer in the structural model, and examination of Fig. 6 ▶(*a*) reveals that this fit is relatively poor. From this mSDDT analysis, the strain was found to be relatively high (32 × 10^−3^) while the long-range-order parameter (*E*) had a near-dynamic value of 0.9 and the best-fit value of Δ_M_ was 5.7′′. The experimental scan from the thickest sample of this set (Fig. 6 ▶ *b*) displayed little evidence of dynamical behavior, and not surprisingly, the static Debye–Waller factor was very small (*E* = 0.01). However, the fit also returned a strain that was surprisingly large (22 × 10^−3^) for a thick layer that one might presume would be significantly relaxed; the best-fit value of Δ_M_ was found to be 5.5′′.

## 4. Discussion

The above results suggest that the mSDDT may be a useful tool for characterizing defective layered structures that are inaccessible to conventional methods based solely on the physics of perfect crystal dynamical diffraction. As mentioned earlier, the experimental plan was to use a set of model samples with defined characteristics in order to benchmark the performance of the mSDDT approach. Such an approach is commonly used in the development of metrology tools, where the analysis of well defined materials is used to validate a new measurement technology. Additionally, it is desirable to use complementary analytical approaches to further confirm the results gained *via* an unproven approach; we have chosen TEM for this purpose.

**Figure 7:**
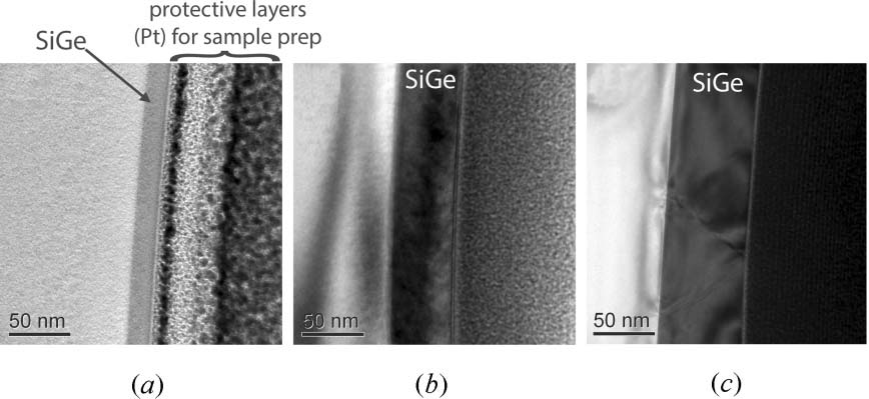
Cross-section TEM images from the constant-composition samples. (*a*) SiGe-a; (*b*) SiGe-b; (*c*) SiGe-c.

Fig. 7 ▶ shows a series of cross-section TEM images that were recorded from the constant-composition sample set (SiGe-a,-b and -c). The samples were prepared for imaging using a focused ion beam process; consequently the micrographs show the platinum layer that was used as a protection layer during the sample preparation, in addition to the SiGe layer and the silicon substrate.

The micrograph of the thinnest layer (SiGe-a) is unremarkable in that the SiGe appears defect-free. The thickness was determined to be 18.0 (5) nm, a value that agrees well with the dynamical fits (both mSDDT and *LEPTOS*) of the X-ray data. More intriguing is the TEM micrograph of sample SiGe-b, where the measured thickness was 47.3 (15) nm, in close correspondence with the mSDDT analysis. This sample appears defect-free and does not show any threading dislocations, although this observation is based solely on cross-sectional (rather than plan-view) imaging and thus may not be sensitive to the presence of non-threading misfit dislocations. The micrograph of the thickest sample (SiGe-c) produced a layer thickness measurement of 66.2 (7) nm, a value that again correlates reasonably well with the presumed 70 nm layer thickness used for the mSDDT model structure.

In addition, the micrograph from SiGe-c shows visible threading dislocations; estimates of dislocation density range from 7.3 × 10^9^ cm^−2^ (top surface area calculation) to 1.06 × 10^12^ cm^−2^ (length/volume calculation). Ultimately, of course, it would be desirable to relate the dislocation density directly to either the static Debye–Waller factor (*E*) or the mosaic spread (Δ_M_). In the case of fully kinematic scattering, expressions for the correlation function for an array of misfit dislocations are available (Pietsch *et al.*, 2004 ▶), although it is not clear how effectively a kinematic treatment could be integrated into a semi- to fully dynamical treatment such as the mSDDT.

The micrographs of the thickest samples (SiGe-b and SiGe-c) showed that, in addition to the SiGe layer itself, there is evidence for strain in the vicinity of the Si/SiGe interface as indicated by a non-uniform intensity distribution. This observation suggests that the use of a single-layer structural model may not be the optimum choice for the mSDDT fitting process. The use of a lamellar model with multiple layers is a common practice in the fitting of HRXRD data *via* dynamical simulation, particularly when a single layer cannot generate an acceptable fit to the data. Under these conditions it is typically assumed that the true sample structure is more complex than a simple single layer, so the use of a more complicated multi-layer model structure is justified.

**Figure 8:**
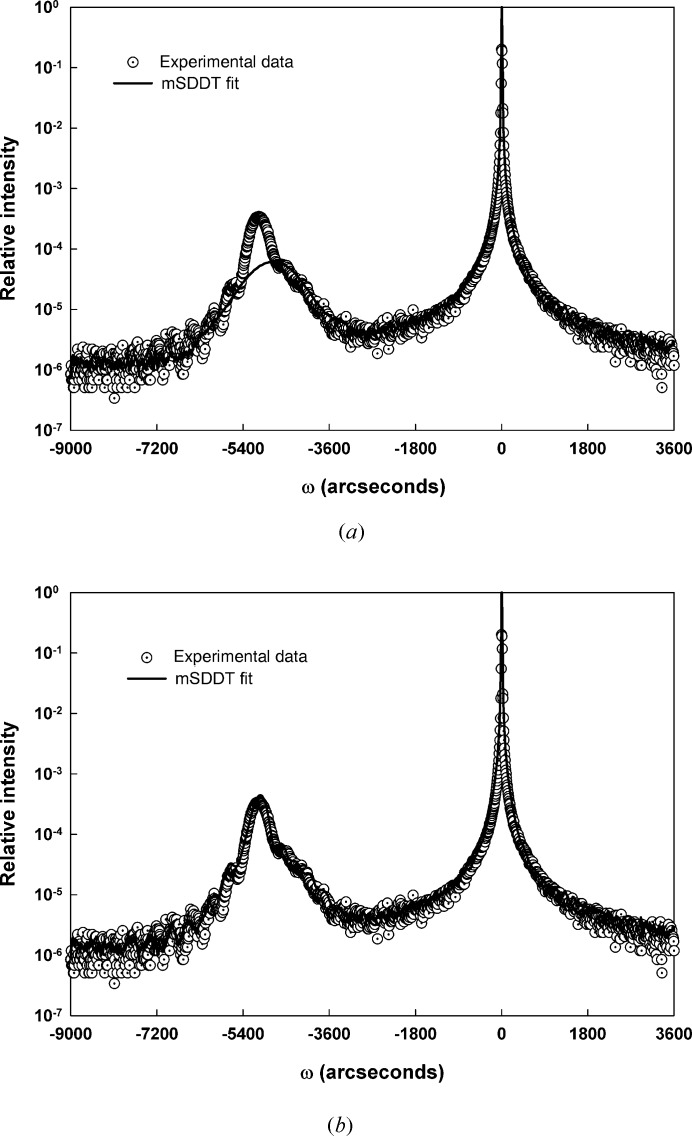
mSDDT fits of SiGe-b: (*a*) interfacial layer only, (*b*) two-layer fit.

Because of the potential impact of an interfacial strain region shown in the TEM data, we returned to sample SiGe-b to examine the impact of using a more complex structural model. Fig. 8 ▶ shows two results. In the first (Fig. 8 ▶ *a*), a thin, highly strained (33 × 10^−3^) and defective (*E* = 0.6) Si layer was presumed to be the sole thin film on the silicon substrate. The mSDDT fit to the data is obviously still poor, but this trial does indeed suggest that the kinematic contributions to the layer peak in the experimental data can be rationalized by a defective interfacial layer. Next, a thick (48 nm) relatively perfect SiGe layer (*E* = 0.95) was combined with the interfacial layer and the resultant calculated mSDDT curve is shown in Fig. 8 ▶(*b*). Inspection shows that the fit has improved considerably, particularly in the vicinity of the layer peak.

These results suggest that the mSDDT may be useful for the model-based fitting of defective thin-film heterostructures. There are, however, issues that need to be resolved before the statistical theory or its variants could be used as a routine tool for applications such as semiconductor metrology. For instance, mention was made earlier of the fact that, in this study, the relationships between parameters such as the static Debye–Waller factor and the mosaic spread (*E* and Δ_M_) are currently not well linked in detail to the physical nature of defects in real materials. If such a correspondence could be achieved, the scope of materials problems that could be addressed using techniques such as the mSDDT would probably be enlarged considerably.

A separate issue involves the sensitivity of the fitting process to parameters such as *E* and Δ_M_: currently we have relatively little knowledge of the ranges that these parameters can attain while still generating an acceptable fit. Closely tied to this is the question of the uniqueness of a fit generated by the statistical diffraction theory, as well as the likelihood that the mSDDT fit variables may be correlated with more common parameters such as layer thickness and composition. Although it is likely to be a difficult task, the successful resolution of these issues should help broaden the applicability of methods based on the statistical dynamical theory to a wider range of materials characterization problems.

Finally, it is worth noting that the thicknesses of the samples investigated in this study are far less than the extinction distance (typically several micrometres) that defines the dynamical coupling between the incident and diffracted wavefields. However, it is well known (Bartels *et al.*, 1986 ▶) that the transition from dynamical to kinematical diffraction is indicated by a continuous decrease in the influence dynamical interactions, such as a reduction in multiple reflections. Hence an analysis of the diffraction characteristics that accompany a transition from high to low structural perfection in epitaxial materials, *versus* the changes in dynamic coupling that come with changes in layer thickness, may represent a fruitful area of investigation.

## Figures and Tables

**Table 1:** Nominal characteristics of the SiGe sample set

Sample ID	Thickness (nm)	Ge (%)
SiGe-2	40	25
SiGe-3	40	50
SiGe-4	40	75
SiGe-a	20	50
SiGe-b	50	50
SiGe-c	70	50
